# Photosynthesis of subtropical forest species from different successional status in relation to foliar nutrients and phosphorus fractions

**DOI:** 10.1038/s41598-018-28800-4

**Published:** 2018-07-11

**Authors:** Guihua Zhang, Lingling Zhang, Dazhi Wen

**Affiliations:** 1 Key Laboratory of Vegetation Restoration and Management of Degraded Ecosystems, South China Botanical Garden, Chinese Academy of Sciences, Guangzhou, 510650 China; 2 College of Resources and Environment, University of Chinese Academy of Sciences, Beijing, 100049 China; 3 Guangdong Provincial Key Laboratory of Applied Botany, South China Botanical Garden, Chinese Academy of Sciences, Guangzhou, 510650 China

## Abstract

The ecophysiological linkages of leaf nutrients to photosynthesis in subtropical forests along succession remain elusive. We measured photosynthetic parameters (*A*_max_, *V*_cmax_, *J*_max_, PPUE), leaf phosphorus (P) and nitrogen (N), foliar P fractions and LMA from 24 species (pioneer, generalist, and climax). *A*_max_ was significantly related to N and P for the pooled data, while significant relationship between *A*_max_ and P was only found in climax species. The mixed-effect model including variables (N, P, and SLA or LMA) for predicting *V*_cmax_ and *J*_max_ best fitted but varied remarkably across succession. Climax species had higher N: P ratios, indicating an increasing P limitation at later succession stage; photosynthesis, however, did not show stronger P than N limitations across all species. Nevertheless, climax species appeared to increase nucleic acid P allocation and residual P utilization for growth, thereby reducing the overall demand for P. Our results indicate that the scaling of photosynthesis with other functional traits could not be uniform across succession, growth variables (e.g. photosynthesis) and species trait identity (e.g. successional strategy) should be considered in combination with N: P ratio when we investigate P limitation in subtropical forests, and variations in P allocation state further influencing photosynthetic rates and P-use efficiency.

## Introduction

Nutrient limitation to primary productivity is widespread in most terrestrial ecosystems globally, and low levels of nitrogen (N) and phosphorus (P) commonly limit or co-limit plant growth and rates of photosynthesis^1,2^. P limitation generally occurs in lowland tropical forests, is particularly strong for sites in Panama and the Amazon basin^3^, while N limitation often occurs in temperate and boreal regions^4–6^. Leaf N:P ratio in terrestrial plants generally serves as a simple and useful indicator of nutrient limitation to primary productivity^7–9^. In forest ecosystems, leaf N:P ratios >16^10^ or 20^8^ usually indicate P limitation. However, P limitation has received comparatively less attention than N limitation, few studies have compared the limitations of leaf P and N concentration on photosynthesis of tropical forest species^11^.

As we all know, the maximum net assimilation rate (*A*_max_) is strongly affected by various leaf traits, for example, leaf thickness^12,13^, leaf mass per area (LMA)^14–16^, and leaf nutrient concentration^15,17,18^. A number of studies have explored how low leaf nutrient concentrations affect leaf photosynthetic capacity in the tropics, particularly for P^6,19–22^. Phosphorus limitation might be manifested in limiting ribulose-1,5-bisphosphate (RuBP) regeneration as the underlying control over *A*_max_ in leaves^23,24^. Previous studies in nutrient-poor ecosystems have shown N limitation can induce larger proportion of leaf N allocated into cell walls, which increases LMA; an increase LMA reduces the photosynthetic N-use efficiency (PNUE) by decreasing *A*_max_^25,26^. However, the ecophysiological linkages between P and *A*_max_ and the underling mechanisms of P limitation are still poorly known^27^.

In the photosynthesis model proposed by Farquhar *et al*.^28^, ‘the maximum carboxylation rate’ (*V*_cmax_) and ‘the maximum electron transport rate’ (*J*_max_) were used to express photosynthetic capacity, which are generally positively related to N, P and specific leaf area in tree leaves^11,21,29^. Various studies have shown that leaf photosynthetic characteristics correspond with successional status^30–32^. The early successional (pioneer) species, which are usually fast-growing and light-demanding, make a greater fractional investment in leaf traits that maximize photosynthetic capacity than late successional (climax) species^30,31,33^. In contrast, leaves of climax species are often have a longer lifespan, higher LMA, chlorophyll to N ratios and lower photosynthetic capacity than leaves of pioneer species^33,34^. As a result, the light-demanding pioneer species are gradually replaced by shade-tolerant climax species. Unlike pioneer species, which occur early in succession, and climax species, which occur late in succession, generalist species can occur throughout succession. What is less clear is how P limitation, which is common in tropical forests, might affect the relationships of photosynthetic capacity to N and LMA during succession in subtropical forests.

Previous studies have shown that plants generally reduce their foliar P concentration in response to low P availability in tropical soils^35,36^. Kedrowski^37^ developed a successful fractionalization scheme based on differential solubility or hydrolysis for the extraction and analysis of various P-containing fractions from plant material. Using a trichloroacetic acid (TCA) extraction method, Close and Beadle^38^ reported differences in the concentrations of insoluble P and inorganic P among plant species. More recently, Hidaka and Kitayama^39^ divided foliar P into four fractions: structural P (lipid P, phospholipids of membranes), metabolic P (including Pi and easily soluble P-containing metabolites), nucleic acid P (RNA and DNA), and residual P (phosphoproteins and unidentified residue). It has been shown that tree species with high photosynthetic P-use efficiency (PPUE) on P-poor sites reduce the demand for foliar P by reducing concentrations of both metabolic P and nucleic acid P^39,40^. However, how plants allocate P among foliar P fractions and how plants develop adaptive strategies to efficiently use P in subtropical region remain unclear.

Plants are probably P-limited in most soils of China, because of the low soil available P content^41^. Insufficient P has become the limiting factor of ecosystem primary productivity and other ecosystem processes in subtropical forests of China^42–44^; however, these studies mainly focused on plant N: P ratios, the real demand of leaf P for growth (e.g. photosynthesis) and its functional partitioning have not been concerned. To our knowledge, few data are available describing the photosynthetic parameters of the subtropical species. Here, we examined how leaf N, P, LMA (or SLA), and foliar P fractions affect photosynthetic performance along a subtropical forest succession. We tested the following hypotheses: (1) Average leaf trait values decrease with succession, pioneer species have higher mean P, N, and photosynthetic parameters (*A*_max_, *V*_cmax_, *J*_max_ and PPUE) than generalist and climax species; (2) Leaf P has a stronger influence over photosynthetic capacity (*A*_max_, *V*_cmax_, and *J*_max_) than N, in particular at later succession stage; and (3) Foliar P fractions change substantially with succession in that climax species, unlike pioneer species, optimize the allocation of P among foliar P fractions in order to maintain their growth and to reduce the overall demand for P.

## Results

### Comparison of leaf traits among successional status

**Figure 1:**
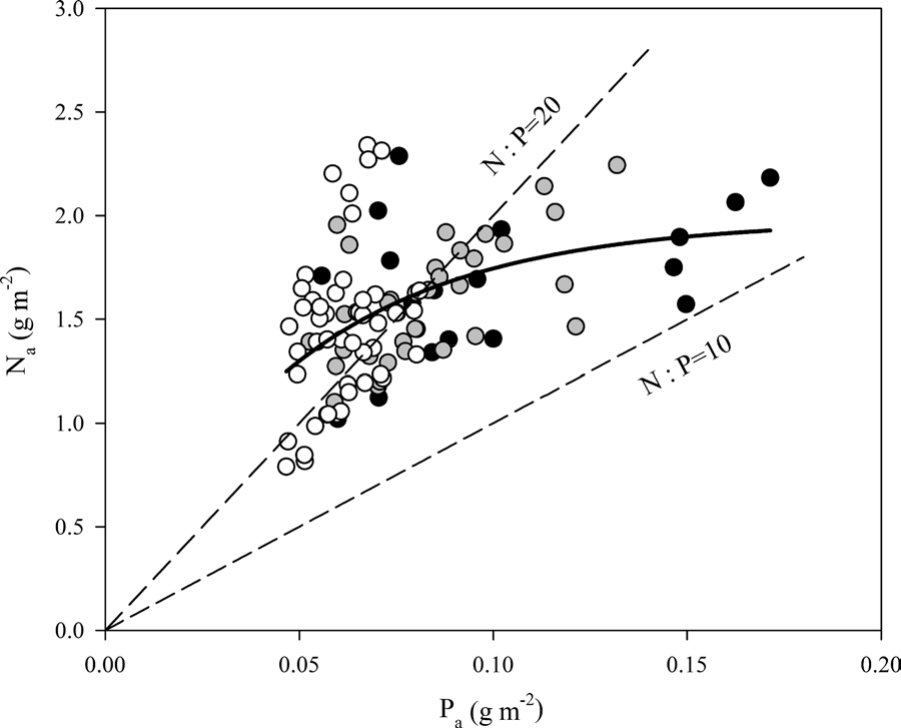
The relationship between area-based leaf P concentration (P_a_) and leaf N concentration (N_a_). Data points represent individual leaf values (19, 37, and 47 individuals from pioneer, generalist, and climax species, respectively). Dashed lines represent the N:P ratios of 10 and 20. Points above the N:P = 20 indicate P limitation; points below the N:P = 10 line indicate N limitation; and points between the lines indicate co-limitation. Symbols: pioneer species (solid); generalist species (grey); climax species (open).

Leaf P concentration of the pioneer species was significantly higher than that of the generalist and climax species, on both an area and a mass basis (both *P* < 0.001; Table 1 and Supplementary Table S1). Pioneer species also exhibited the highest N_a_ values, although no significant differences in N_a_ were observed among successional groups (*P* = 0.08, Table 1). The pioneer and climax species leaves exhibited similar values for N_m_; however, the climax species leaves had the highest N:P ratio (*P* < 0.001, Table 1). Given that a leaf N:P ratio >20 generally indicates P limitation as opposed to N limitation^8^, P limitation was evident in 21% of pioneer species, 38% of generalist species, and 66% of climax species (Fig. 1). On the basis of both area and mass, all photosynthetic parameters (*A*_max_, *V*_cmax_, *J*_max_ and PPUE) significantly differed among species with succession (*P* < 0.01; Table 1), i.e., these values were greater for pioneer species than for generalist and climax species. *J*_max_/*V*_cmax_ ratio did not significantly differ among successional groups (*P* = 0.525, Table 1).

**Table 1:** Area-based leaf traits for the subtropical forest species in three successional groups.

Group	N_a_	P_a_	N:P ratio	LMA	*A*_max,a_	*V*_cmax,a_	*J*_max,a_	*J*_max,a_/*V*_cmax,a_	PPUE
	(g m^−2^)	(g m^−2^)		(g m^−2^)	(µmol m^−2^ s^−1^)	(µmol m^−2^ s^−1^)	(µmol m^−2^ s^−1^)		(µmol mol^−1^ s^−1^)
Pioneer	1.65 ± 0.08 b	0.10 ± 0.01 c	18.28 ± 1.40 a	86.06 ± 8.73 b	12.43 ± 0.93 c	69.08 ± 4.86 b	88.72 ± 7.31 c	1.29 ± 0.05 a	5222.20 ± 520.82 b
Generalist	1.58 ± 0.05 ab	0.08 ± 0.01 b	19.99 ± 0.69 a	92.54 ± 2.37 b	9.92 ± 0.57 b	56.98 ± 3.11 a	73.42 ± 3.91 b	1.31 ± 0.04 a	5052.24 ± 367.63 b
Climax	1.46 ± 0.05 a	0.06 ± 0.01 a	23.92 ± 0.88 b	70.50 ± 2.48 a	6.54 ± 0.28 a	49.51 ± 2.00 a	60.84 ± 2.40 a	1.25 ± 0.03 a	3899.78 ± 166.75 a

Values shown are group averages (±SE). Means in a column followed by different letters are significantly different (*P* < 0.05). N_a_, leaf nitrogen concentration; P_a_, leaf phosphorus concentration; leaf N:P ratio, leaf nitrogen to phosphorus ratio; LMA, leaf mass per area; *A*_max,a_, maximum photosynthesis assimilate rate; *V*_cmax,a_, maximum carboxylation velocity; *J*_max,a_, maximum electron transport rate; J_max;a_/V_cmax;a_, ratio of maximum carboxylation velocity over maximum rate of electron transport; PPUE, photosynthetic P-use efficiency.

### Bivariate relationships between photosynthesis and leaf traits

For all data pooled, leaf N_a_ and P_a_ were highly and positively correlated (r^2^ = 0.21; *P* < 0.001, Fig. 1). Across all 24 species, N_a_ exhibited a weak, positive correlation with LMA (Supplementary Fig. S1a, r^2^ = 0.13; *P* < 0.01). SMA tests for common slopes revealed a significant difference among the different successional status (Supplementary Table S2); the y-axis intercept of the relationship was higher for climax species, indicating that the climax species might have a higher N_a_ for a given LMA than pioneer species. Compared to N_a_, P_a_ showed a stronger relation with LMA (Supplementary Fig. S1b, r^2^ = 0.34; *P* < 0.001). Additionally, P_a_ for a given LMA was lower for climax species than pioneer species (Supplementary Table S2).

**Figure 2:**
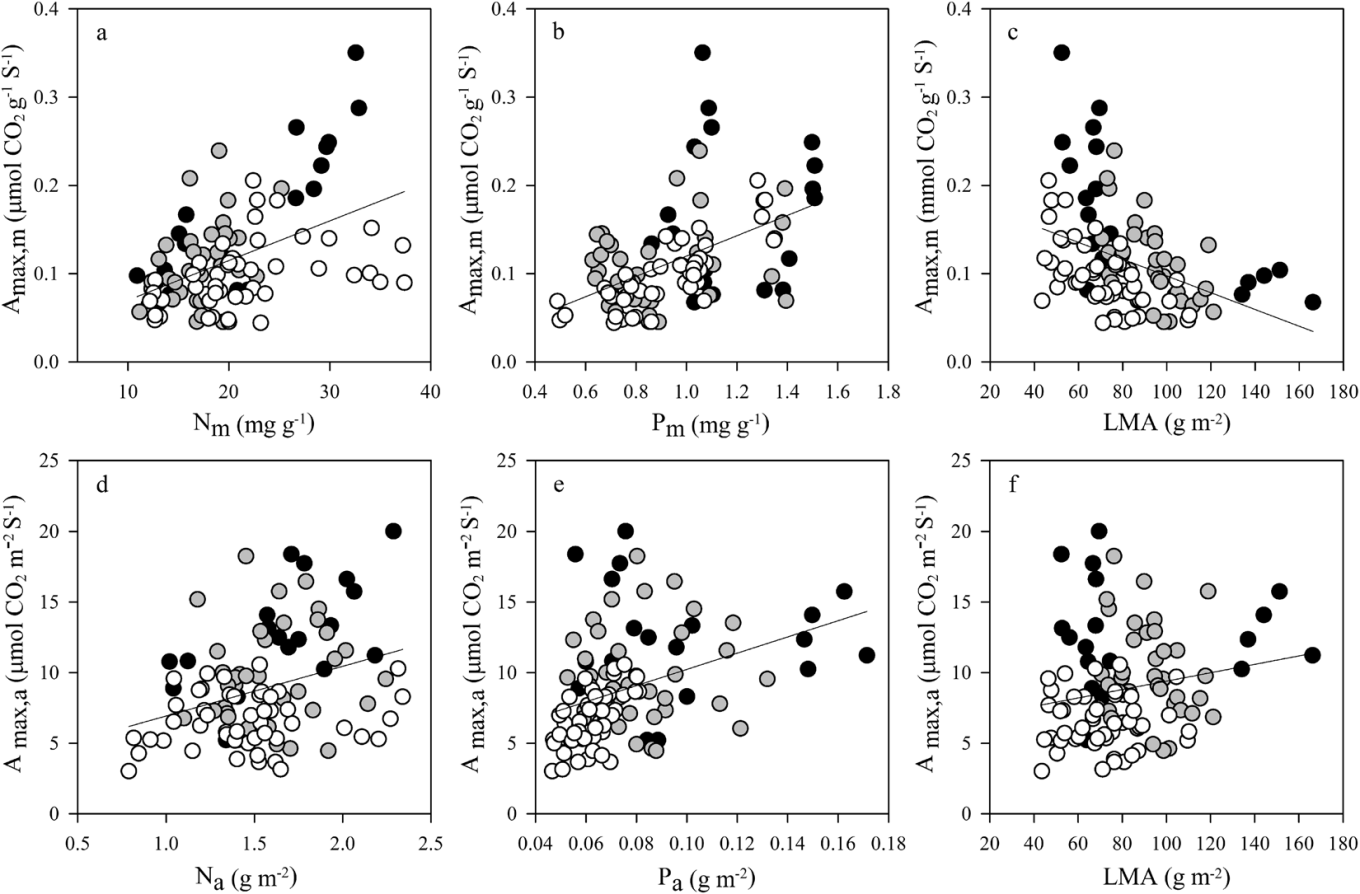
The log-log relationships between photosynthesis capacity (the maximum photosynthesis assimilate rate, *A*_max_) and leaf traits including nitrogen concentration (N), phosphorus concentration (P), and leaf mass per area (LMA). Note that *A*_max_ values are based on measurements of mass in the upper panels and on area in the lower panels. Data points represent individual leaf values (19, 37, and 47 individuals from pioneer, generalist, and climax species, respectively). Standardized major axis (SMA) regressions are presented in Table S3. Symbols are the same as in Fig. 1.

Bivariate relationships of *A*_max_ on N and P (either area-based or mass-based) were highly significant for the pooled data (Fig. 2, *P* < 0.01). When the area-based data were pooled, variations in *A*_max,a_ were slightly explained by variations in P_a_ (*P* < 0.001; r^2^ = 0.19) and N_a_ (*P* = 0.001; r^2^ = 0.10). However, Supplementary Table S3 shows that when the data were grouped by successional status, area-based relationships were significant between *A*_max,a_ and N_a_ for the pioneer species (*P* = 0.012; r^2^ = 0.32) and between *A*_max,a_ and P_a_ for the climax species (*P* = 0.001; r^2^ = 0.31). For all data pooled, LMA was weakly positively related to *A*_max,a_ (*P* < 0.05; r^2^ = 0.04), but this relationship was not significant for any of the three successional status (Supplementary Table S3). When the mass-based data were pooled, *A*_max,m_ was significantly and positively correlated with P_m_ (r^2^ = 0.26), N_m_ (r^2^ = 0.21), and negatively with LMA (r^2^ = 0.15) (all *P* < 0.001). Still, *A*_max,m_ was significantly correlated with N_m_ and LMA for all successional groups, while *A*_max,m_ and P_m_ were poorly related for pioneer or generalist species (Supplementary Table S3).

**Figure 3:**
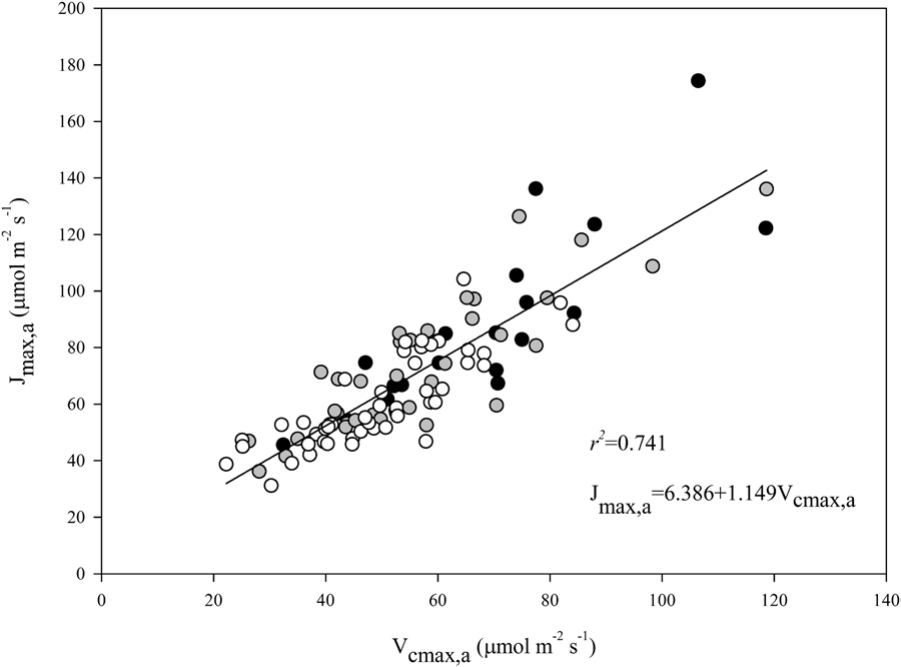
The relationship between area-based maximum carboxylation velocity (*V*_cmax,a_) and area-based maximum electron transport rate (*J*_max,a_). Data points represent individual leaf values (19, 37, and 47 individuals from pioneer, generalist, and climax species, respectively). Standardized major axis (SMA) regressions are given in Table S4. Symbols are the same as in Fig. 1.

**Figure 4:**
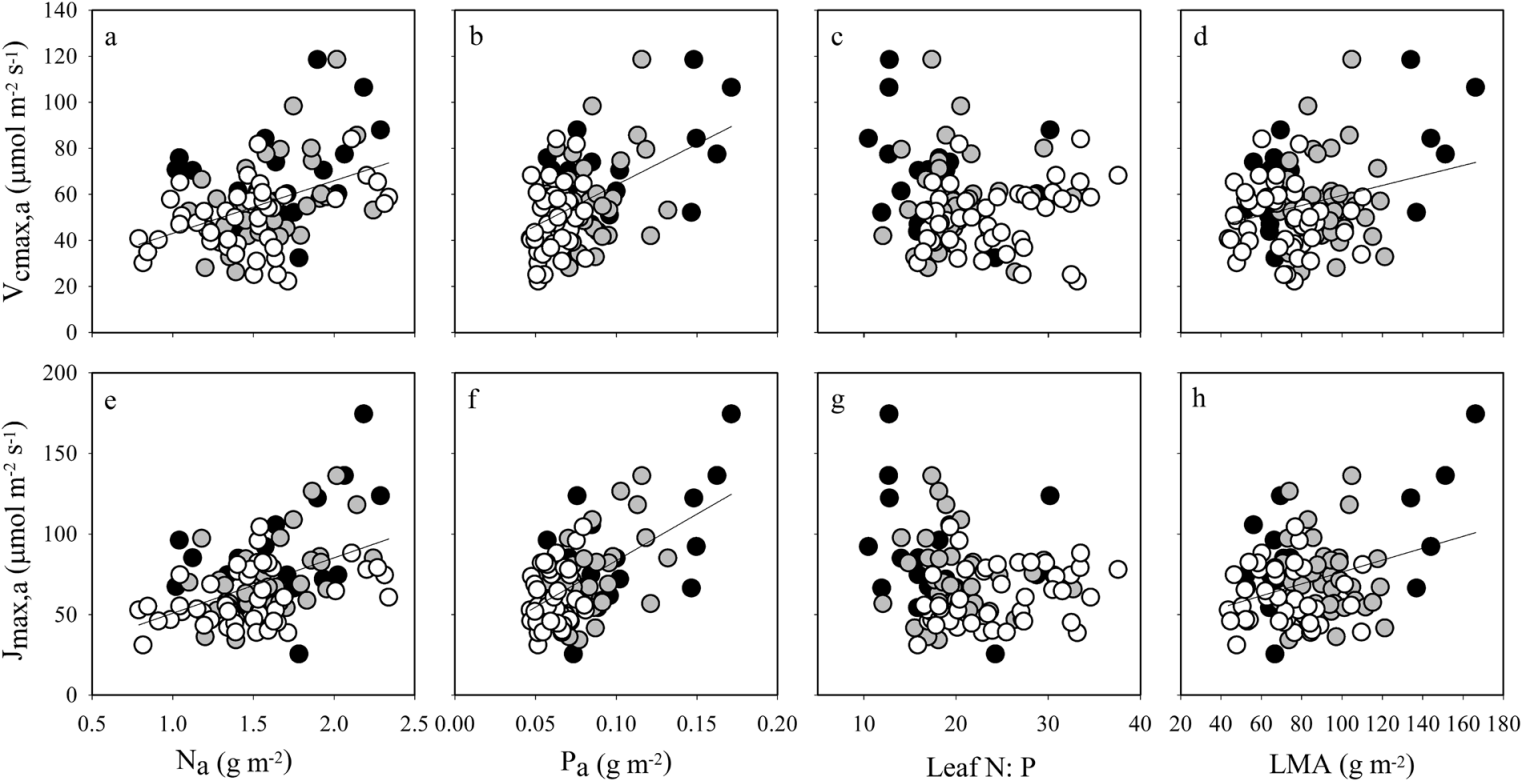
The log-log relationships between area-based photosynthesis capacity and leaf traits including nitrogen concentration (N_a_) (**a**,**e**), phosphorus concentration (P_a_) (**b**,**f**), leaf N:P (**c**,**g**), and leaf mass per area (LMA) (**d**,**h**). (**a**–**d**) Show the maximum carboxylation velocity (*V*_cmax,a_), while e-h show the maximum electron transport rate (*J*_max,a_). Data points represent individual leaf values (19, 37, and 47 individuals from pioneer, generalist, and climax species, respectively). Standardized major axis (SMA) regressions are given in Table S4. Symbols are the same as in Fig. 1.

The regression of *J*_max,a_ on *V*_cmax,a_ using data from all species suggested a very tight co-ordination between the two parameters (*P* < 0.001, r^2^ = 0.74, Fig. 3). Variations in *J*_max,a_ were strongly correlated with *V*_cmax,a_ all for the three successional groups (Supplementary Table S4). Across all species, *V*_cmax,a_ and *J*_max,a_ were correlated with leaf traits, and the bivariate relationships were stronger with P_a_ (*P* < 0.001; r^2^ = 0.17 for *V*_cmax_; r^2^ = 0.23 for *J*_max_) than with N_a_ (*P* < 0.01; r^2^ = 0.13 for *V*_cmax_; r^2^ = 0.18 for *J*_max_) (Fig. 4). No significant relationship was found between *V*_cmax,a_ (or *J*_max,a_) and N:P ratio (Fig. 4). Both *V*_cmax,a_ and *J*_max,a_ were marginally correlated with LMA (*P* < 0.01; r^2^ = 0.07 and r^2^ = 0.06, respectively) (Fig. 3). Within the pioneer group, significant relationship of *V*_cmax,a_ was observed only with LMA (*P* = 0.021, r^2^ = 0. 27), no significant relations with N_a_, P_a_, or N:P ratios (Supplementary Table S4). Within the generalist and climax group, *V*_cmax,a_ was positively related to N_a_ (*P* = 0.002, r^2^ = 0.24 for generalist species; *P* = 0.050, r^2^ = 0.08 for climax species) but not with LMA, P_a_, or N:P ratio (Supplementary Table S5). Similar patterns were observed for *J*_max,a_ (Supplementary Table S4).

### Modelling variations in photosynthetic capacity from leaf traits

We used linear mixed-effect to model variations in photosynthetic parameters (*V*_cmax_ and *J*_max_) (Table 2 and Supplementary Table S5). The regression coefficients (r^2^) ranged from 0.30 to 0.53 and were substantially higher when the variables were expressed on a mass basis than on an area basis. The model’s random variance indicated that species accounted for less than 3% of the unexplained variance for models.

**Table 2:** The linear mixed-effect models, with mass-based (a) and area-based (b) leaf photosynthetic capacity (*V*_cmax_ and *J*_max_) as the response variables, each showing fixed and random effects.

	*V*_cmax_					*J*_max_				
	Fixed effect			Random variance (%)		Fixed effect			Random variance (%)	
	Best model	r^2^	*P*	Species	Residual	Best model	r^2^	*P*	Species	Residual
**(a) Mass-based model**										
All	P_m_ + N_m_*SLA	0.47	<0.01	0.19	99.81	P_m_ + N_m_ * SLA	0.48	<0.01	0.00	100.00
Pioneer	SLA	0.39	<0.01	0.00	100.00	N_m_ * SLA	0.32	<0.01	0.00	100.00
Generalist	N_m_ * SLA	0.41	<0.01	0.00	100.00	N_m_ * SLA	0.50	<0.01	0.00	100.00
Climax	P_m_ + N_m_*SLA	0.53	<0.01	0.00	100.00	N_m_ * SLA	0.49	<0.01	0.00	100.00
**(b) Area-based model**										
All	N_a_ + P_a_ * LMA	0.23	<0.01	0.47	99.53	N_a_ + N_a_ * P_a_	0.36	<0.01	0.20	99.80
Pioneer	LMA	0.19	<0.05	0.00	100.00	LMA	0.27	<0.05	0.00	100.00
Generalist	P_a_ * LMA	0.13	<0.05	0.00	100.00	LMA + N_a_ * LMA	0.31	<0.01	0.00	100.00
Climax	N_a_ + N_a_ * LMA	0.16	<0.01	2.56	97.44	N_a_	0.17	<0.01	0.00	100.00

For the best models, explanatory variables are: leaf nitrogen (N) and phosphorus (P) concentrations; leaf area per mass (SLA) and leaf mass per area (LMA). Species was used as a random component of the model. The best model for *V*_cmax_ and *J*_max_ for all species (All), and for the pioneer species (Pioneer), the generalist species (Generalist) and the climax species (Climax) are shown. The coefficient of variation (r^2^) and significance (*P*) for the linear regression of the modelled vs measured data are also shown and the contribution of random effect to the variance with the dataset.

Based on leaf mass, a combination of leaf N, P and SLA accounted for 47% of the variation in *V*_cmax,m_. Similar to *V*_cmax,m_, variations in *J*_max,m_ were largely explained by a combination of N, P and SLA; the best model explained 48% of the variation in *J*_max,m_. When these analyses were repeated using area-based data, relationships were similar to those described for mass-based measurements. For the pioneer species, the species with the highest N and P, SLA was the important fixed effect for explaining *V*_cmax_ and *J*_max_. For the generalist and climax species, SLA (or LMA) was important and the importance of N and P varied depending on whether data were expressed on a mass or an area basis. Importantly, for both the mass- and area-based mixed-effect models, both N and P are important predictors of *V*_cmax_ and *J*_max_, when data from all species were combined.

### Variation in foliar P fractions

The overall average concentration of each foliar P fraction was significantly higher in pioneer species than in the other two groups (all *P* < 0.05) (Table 3), although the mean concentration of both structural P and nucleic acid P were not different between generalist species and climax species. For each group of species, concentrations tended to be higher for nucleic acid P than for the other fractions (all *P* < 0.05) (Table 3). The mean percentage of P represented by structural P did not differ among the three successional groups (*P* = 0.766). The metabolic P percentage was significantly lower in generalist species than in the other groups (*P* < 0.001). The nucleic acid P percentage was lower in pioneer species than in the other groups (*P* < 0.001). Conversely, the residual P percentage was significantly lower in climax species than in the other groups (*P* < 0.001).

**Table 3:** Concentrations (mg g^−1^) and percentages (%) of foliar P fractions of subtropical forest species in different successional groups.

P fraction	Pioneer		Generalist		Climax		*P* value
Structural P							
Concentration	0.248 ± 0.007	B a	0.204 ± 0.009	BC b	0.206 ± 0.009	C b	*P* = 0.009
Percentage	21.88 ± 1.27	a	22.63 ± 0.45	a	22.49 ± 0.51	a	*P* = 0.766
Metabolic P							
Concentration	0.358 ± 0.022	A a	0.226 ± 0.010	B c	0.271 ± 0.009	B b	*P* < 0.001
Percentage	29.98 ± 0.94	a	25.12 ± 0.44	b	30.15 ± 0.77	a	*P* < 0.001
Nucleic acid P							
Concentration	0.349 ± 0.017	A a	0.288 ± 0.009	A b	0.309 ± 0.013	A b	*P* = 0.021
Percentage	29.47 ± 0.66	b	32.66 ± 0.57	a	33.26 ± 0.48	a	*P* < 0.001
Residual P							
Concentration	0.227 ± 0.020	B a	0.183 ± 0.013	C b	0.129 ± 0.007	D c	*P* < 0.001
Percentage	18.76 ± 0.94	a	19.58 ± 0.68	a	14.07 ± 0.57	b	*P* < 0.001

Values are means ± SE. Within each P fraction, concentrations followed by different lowercase letters are significantly different at *P* < 0.05 among successional groups. Within each P fraction, percentages followed by different by different lowercase letters are significantly different at *P* < 0.05 among successional groups. Within each successional group, concentrations followed by different uppercase letters are significantly different at *P* < 0.05 among P fractions.

Across all 24 species, the concentrations of each foliar P fraction were positively related to P_m_ (Table 4). For regressions of concentrations of P fractions on P_m_, SMA slopes did not significantly differed (*P* = 0.107), while the intercept was significantly higher for the concentration of nucleic acid P than for the concentrations of other fractions (*P* = 0.001, Table 4). But the values for both nucleic acid P and metabolic P  were followed by a lowercase c, indicating that the slopes were not different. The concentration of each foliar P fraction had significant positive relationship with N_m_, and negative relationship with N:P ratio (Table 4). Moreover, except residual P, LMA significantly increased with decreasing P fractions concentration, and the intercept of nucleic acid P for LMA was larger than those of the other two fractions (*P* < 0.001, Table 4). The SMA slopes of regressions of concentration of each foliar P fraction on N_m_, N:P ratio, and LMA did not significantly differ among foliar fraction types (all *P* > 0.1).

**Table 4:** Standardized major axis (SMA) relationships between log-log transformed foliar phosphorus (P) fractions and total leaf P concentration (P_m_), leaf mass per area (LMA), leaf nitrogen concentration (N_m_), and N:P ratio across the three successional groups.

Leaf trait	Structural P	Metabolic P	Nucleic acid P	Residual P	*P*-value
**P**_**m**_					
*r* ^2^	0.630***	0.675***	0.820***	0.568***	
Slope	1.051 (0.810, 1.370)	1.133 (0.828, 1.472)	1.191 (0.980, 1.437)	1.771 (1.346, 2.326)	0.107
Intercept	−0.656 b	−0.546 c	−0.485 c	−0.770 a	0.001
**N**_**m**_					
*r* ^2^	0.124***	0.285***	0.381***	0.036^*^	
Slope	1.044 (0.636, 1.727)	0.858 (0.640, 1.185)	0.937 (0.656, 1.427)	1.764 (1.090, 2.847)	0.311
Intercept	−2.017 b	−1.669 c	−1.722 c	−3.070 a	<0.001
**N:P ratio**					
*r* ^2^	0.145***	0.149***	0.034	0.286***	
Slope	−1.065 (−0.681, −1.813)	−1.263 (−0.831, −1.843)	−1.067 (−0.703, −1.746)	−1.950 (−1.287, −3.038)	0.430
Intercept	0.713 a	1.081 c	0.872 b	1.741 d	<0.001
**LMA**					
*r* ^2^	0.319***	0.325***	0.400***	0.016	
Slope	−1.116 (−0.791, −1.671)	−1.160 (−0.766, −1.789)	−1.244 (−0.908, −1.741)	−1.987 (−1.246, −3.323)	0.370
Intercept	1.438 a	1.631 ab	1.838 b	2.962 c	<0.001

Analysis undertaken using species replicates. For each leaf trait, intercepts followed by different letters indicate significant differences among P fractions. *, **, and *** indicate significance at *P* < 0.05, <0.01, and <0.001, respectively.

### Relationships between foliar P fractions and photosynthetic rates and P-use efficiency

**Figure 5:**
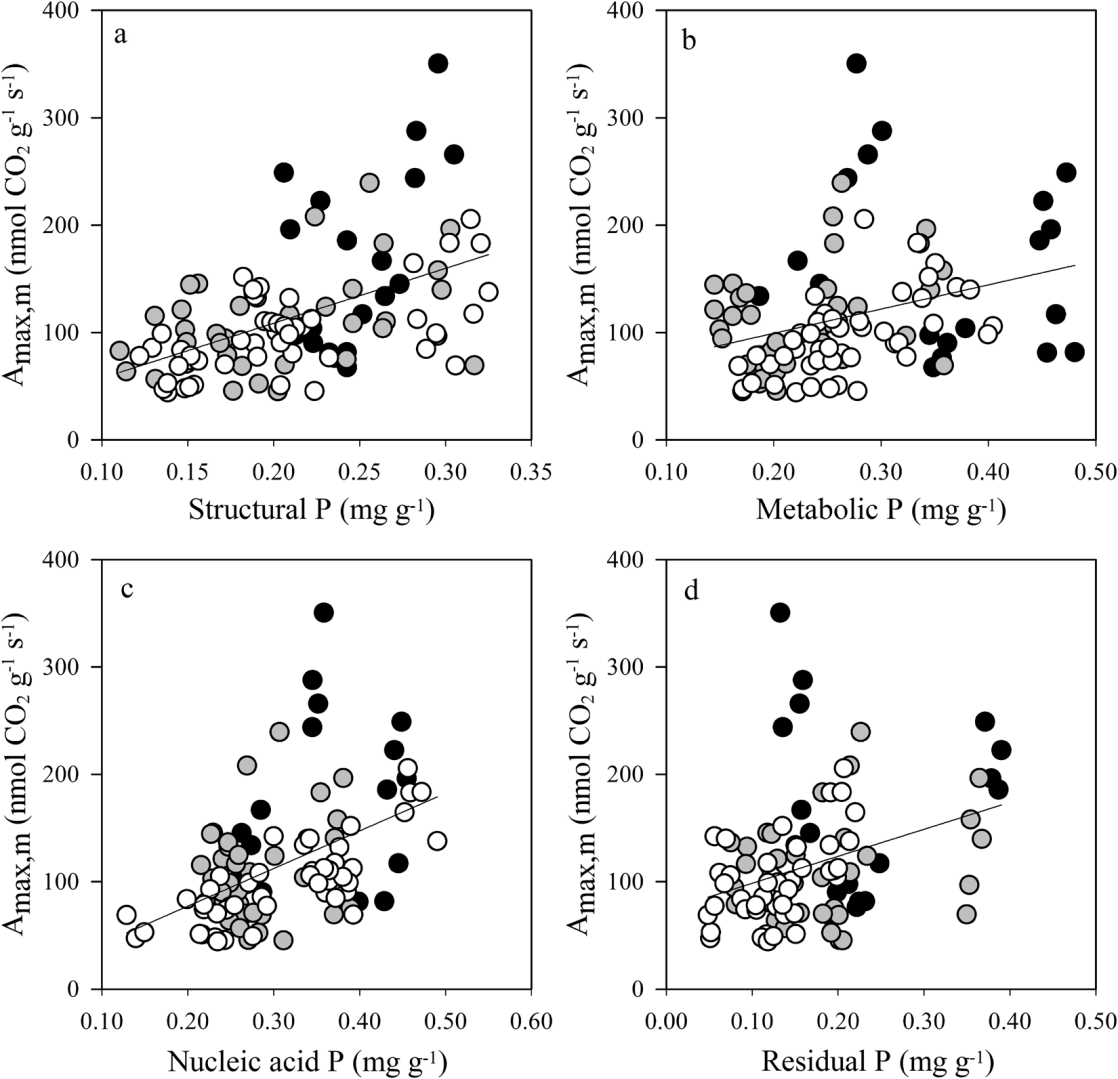
The relationships between the mass-based maximum photosynthesis assimilate rate (*A*_max,m_) and the concentration of four foliar P fractions (structural P, metabolic P, nucleic acid P, and residual P). Data points represent individual leaf values (19, 37, and 47 individuals from pioneer, generalist, and climax species, respectively). Symbols are the same as in Fig. 1.

**Figure 6:**
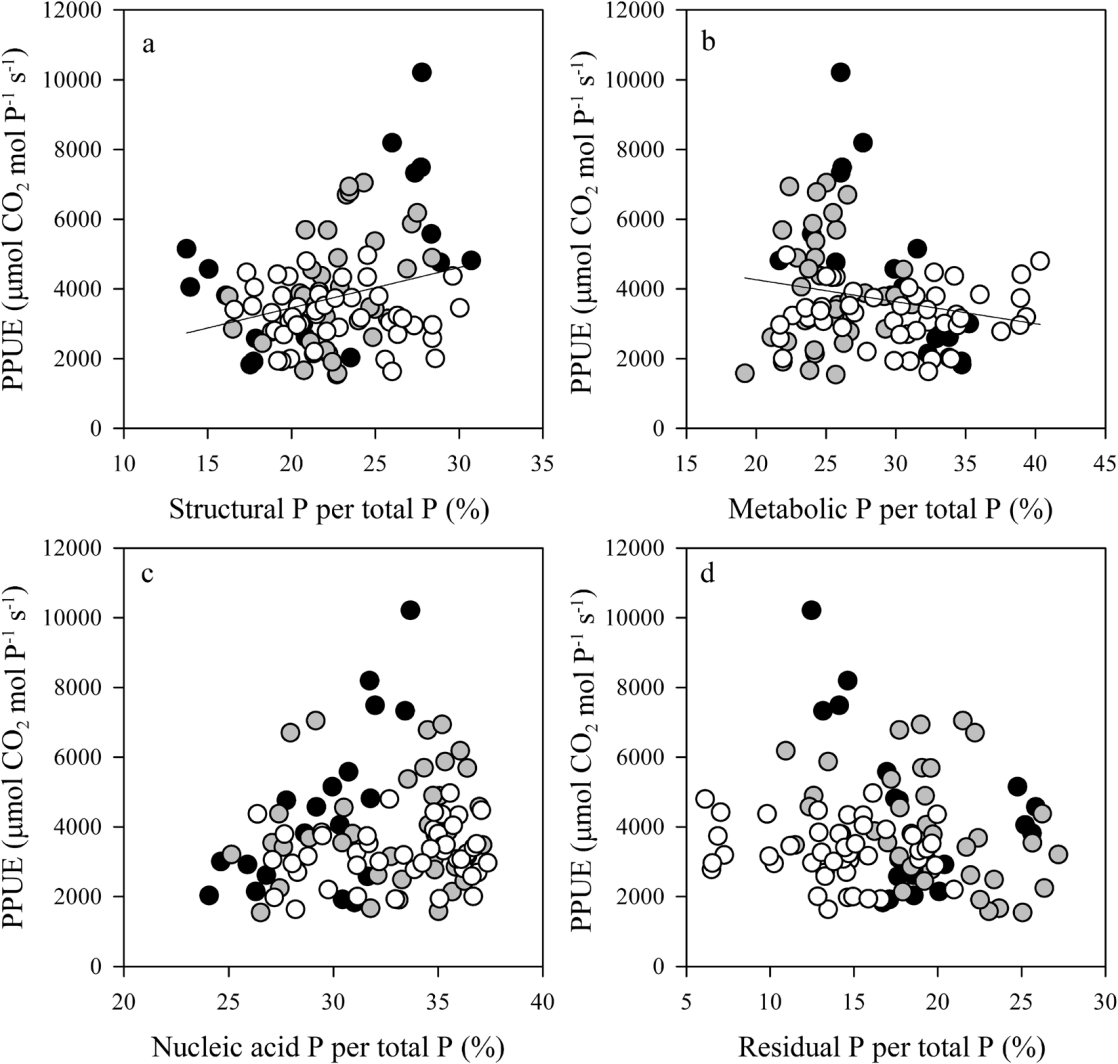
The relationships between photosynthetic P-use efficiency (PPUE) and the percentage of four foliar P fractions (structural P, metabolic P, nucleic acid P, and residual P). Data points represent individual leaf values (19, 37, and 47 individuals from pioneer, generalist, and climax species, respectively). Symbols are the same as in Fig. 1.

Among all 24 species, *A*_max,m_ was positively correlated with the concentration of foliar P fractions (Fig. 5). The coefficients of determination between *A*_max,m_ and P fraction concentrations (r^2^ = 0.26 for structural P and 0.27 for nucleic acid P) were similar to that between *A*_max,m_ and P_m_ (r^2^ = 0.26). The correlation coefficients between *A*_max,m_ and metabolic P (r^2^ = 0.11) and residual P (r^2^ = 0.10) were low and positive. PPUE was positively correlated with the percentage of structural P (r^2^ = 0.07, *P* < 0.01; Fig. 6a) but was negatively correlated with the percentage of metabolic P (r^2^ = 0.03, *P* < 0.05; Fig. 6b). PPUE was not correlated with the percentage of nucleic acid P or residual P (Fig. 6c,d).

## Discussion

The first hypothesis was supported, and overall average area-based N_a_, P_a_, and photosynthetic parameters (*A*_max_, *V*_cmax_, *J*_max_, PPUE) were higher in pioneer species than in generalist and climax species (Table 1), indicating the photosynthesis capacity with accompanying P use efficiency generally declined with succession proceeding. These results were consistent with what is known about photosynthetic physiology, such as, photosynthetic capacity decreases along the successional axis^30,31,33,34^. Rijkers *et al*.^45^ found that early-successional species had higher SLA than that of late-successional species in a lowland tropical area of French Guiana. However, no clearly consistent pattern of LMA variations along with succession was found in the present study, i.e. mean LMA values were higher for the generalist species than for the pioneer and climax species (Table 1). This result might be due to the large variations in LMA among species within the same successional group.

Our results showed that leaf N:P ratios ranged from 10.5 to 37.6 across the 24 species, and 48% of the species measured were limited by P and the remainder were co-limited by N and P (Fig. 1). The relatively high values for N:P ratios reported here agree with our prior knowledge that these systems are P limited^42,44^. Moreover, the climax species had the highest N:P ratio (Table 1), showing that P limitation is more pronounced in climax species. However, some studies reported that leaf N:P ratios are not definitive indicators of N or P limitation^46,47^. In a study in Australia, for example, median N:P ratios were relatively high (>20) at all sites, but evidence did not indicate that photosynthesis was limited by P for either forest or savanna trees^48^. These inconsistencies between N:P ratios and P limitation may result from the great variability of N:P ratios throughout biomes and among and within species, and although analogous biogeochemical constraints may exist for individual plant organs, differences can vary by up to an order of magnitude^8,49–51^. For this reason, we also explore how leaf N and P concentration in these subtropical systems may affect photosynthetic biochemistry.

Several studies have reported that positive relationships between leaf N (or P) and photosynthetic rates^18,19,30,52^. Indeed, *A*_max_ was positively related to N and P, and these relationships were evident whether measurements were mass-based or area-based (Fig. 2). The slopes of the relationships between *A*_max_ and key leaf traits (N, P, and LMA) differed depending on the successional status, more importantly, significant relationship between *A*_max_ and P was only found in the climax species (Supplementary Table S3). The climax species had both the lowest P and the lowest *A*_max_ values. This result suggests that a reduction in the total P is a main factor limiting photosynthesis of climax species, in agreement with studies related low *A*_max_ in leaves with P limitation^24^. The relationship between *A*_max_ and N may be constrained by low P in P-limited ecosystems^18,27^, out results highlight that the slope of *A*_max_ on N was shallower for the climax species (with low P values) than for the pioneer species (with high P values). Further, *A*_max_ was negatively correlated with LMA when *A*_max_ was based on mass measurements (Fig. 2f). This is in line with an increase in LMA leading to an increase in resistance to CO_2_ diffusion within the leaf and eventually to a decrease in *A*_max_^53,54^.

In this study, *J*_max_ and *V*_cmax_ were closely related, and the linear regression had a slope of 1.15 ± 0.07 (Fig. 3). A larger common slope of *J*_max_ and *V*_cmax_ was 1.27 when the SMA test was used. The slope in our study was less than those from global analyses (1.64 in Wullschleger^55^, 1.67 in Medlyn *et al*.^56^). Furthermore, the slope of the *J*_max_-*V*_cmax_ relationship was steeper for pioneer species than for generalist and climax species in the current study (Supplementary Table S4). This discrepancy may be explained by the relative high light conditions experienced by the pioneer species.

The photosynthetic capacity (*V*_cmax_ and *J*_max_) of trees is generally positively related to leaf P concentration, especially under conditions of P limitation^6,20,21,48,57^. In this study, we demonstrated that across all 24 species, *V*_cmax,a_ and *J*_max,a_ were significantly and positively related to N_a_ and P_a_ and weakly and negatively related to the N:P ratio (Fig. 4). When data from all 24 species were modelled, the line mixed-effect modeling analysis performed here demonstrated that, both N and P were important as variables for predicting *V*_cmax_ and *J*_max_, whether measurements were area-based or mass-based (Table 2). Additionally, leaf P and the interaction between N and SLA have proven to be significant explanatory variables in mixed-effect models of variations in photosynthetic capacity, explaining about 50% of the observed variations in *V*_cmax,m_ and *J*_max,m_ (Table 2a). The best combination of variables (N, P, and SLA or LMA) for modelling *V*_cmax_ and *J*_max_ changed with succession. We also found that species played a very weak role in modelling photosynthetic capacity, and therefore that limitations to photosynthetic capacity were likely to be results of environmental factors. In addition, these differences in the best mixed-effect model were in line with the observed variation in the relationships of photosynthetic capacity (*V*_cmax_ and *J*_max_) with leaf traits (N, P, and LMA) from SMA regression analysis (Supplementary Table S4). Collectively, these results show that N, P, and LMA or SLA, and especially N and P, help explain the variations in *A*_max_, *V*_cmax_ and *J*_max_ in this study and that the degree of influence for different trait of three successional groups are different.

Taken together, the second hypothesis that P has a stronger influence over photosynthetic capacity than N, was only supported in the climax species, but was not supported in the other two species groups; leaf N and P are the best predictors of photosynthetic capacity. These results highlight the importance for the consideration of not solely N:P ratio but also growth variables (e.g. photosynthesis) and species identity (e.g. successional strategy) into nutrient limitation determination in the highly species-rich and diversified subtropical forest ecosystems.

In our study, the concentrations and the percentages of total P represented by functional P fractions were higher for nucleic acid P than for other foliar P fractions except for metabolic P in the pioneer species (Table 3). These results are in line with past studies showing that nucleic acid P is the largest pool throughout the growing season^39,58^. A previous study suggested that a reduction in foliar P concentration is strongly correlated with a reduction in the concentrations of both metabolic P and nucleic acid P with decreasing soil P availability^39^. Similarly, our results indicate that reductions in the concentrations of both nucleic acid P and metabolic P explain most of the reduction in leaf P concentration among the three successional groups (Table 4). Another previous study showed that the concentration of structural P is greater in fast-growing plants with small LMA than in slow-growing plants with large LMA^59^, which was partial in line with the results of the present study. The pioneer species and climax species exhibited similar values for LMA, however, pioneer species had higher structural P concentration (Table 4). Moreover, our results show that the concentration of each foliar P fraction decreases along the successional axis, and that the climax species have a greater nucleic acid P percentage and a lower residual P percentage than pioneer and generalist species (Table 4). Overall, these findings suggest that there are trade-offs in the P allocation among nucleic acid P and metabolic P with residue P.

*A*_max,m_ positively correlated with P_m_ and the concentrations of each foliar P fraction (Figs 2b and 5). Mean *A*_max,m_ values and mean concentrations of P_m_ and of each foliar P fraction were lower in climax species than in pioneer species (Table 3 and Supplementary Table S1). As a result, climax species had lower mean PPUE values than pioneer species in this study, which agrees with previous studies of tropical rainforest trees^31^. PPUE was positively correlated with *A*_max,m_ (r^2^ = 0.68, P < 0.001) and weakly and negatively correlated with P_m_ and LMA (data not shown). Hidaka and Kitayama^40^ found that PPUE was negatively correlated with the proportion of structural P but tended to be positively correlated with the proportion of metabolic P. In contrast, we found that PPUE was weakly and positively correlated with the structural P percentage and negatively correlated with the metabolic P percentage (Fig. 6). Hence, the lower PPUE values in climax species can be explained by reduced photosynthetic activity. Finally, the third hypothesis was supported by our results - the climax species may increase their nucleic acid P percentage to maintain their growth and decrease residual P percentage to reduce their demand for P (i.e., reducing total leaf P concentration) as adaptation strategies to soil available P impoverishment at later succession stage.

Previous studies were restricted to Alaskan tree species on P-rich soils^58,60^ or to Mount Kinabalu plant species on P-poor sites^39,40^. Our studies on foliar P fractions and the relationships between foliar P fractions and leaf traits in species representing successional strategies can improve our understanding of what the magnitude species are adapted to low soil P availability and how forests can maintain their high productivity in the highly weathered and acidified soils in the subtropics. We conclude that predicting future dynamics of forest ecosystems in response to global change requires a better understanding of the variations of nutrient limitation, not solely on the base of plant N: P ratios but also by incorporating growth variables (e.g. photosynthesis), and in particular the P adaptation strategies created by ecosystem succession of the subtropical forests.

## Materials and Methods

### Study site and plant materials

This study took place at the Dinghushan Biosphere Reserve (DBR) in central Guangdong Province, southern China (21°09′21′′–21°11′30′′ N, 112°30′39′′–112°33′41′′E). The region is characterized by a typical subtropical monsoon climate, with mean annual temperature is 21.4 °C, and annual average precipitation is 1927 mm with 80% occurring during the wet season (April to September). The soils are classified as Ultisol and Udult according to the USDA soil classification. A total of 24 species across successional stages were sampled in this study (Supplementary Table S6). Three to five individuals were selected for each species. The studied species are common and typical in each stage of the succession according to the long-term forest community studies of the reserve^61,62^. We used long-reach pruner to collect middle canopy branches (supporting leaves considered to be typically exposed to full sunlight for much of the day) from tall plant species. The detached branches had been recut under water immediately after harvesting to preserve xylem water continuity, prior to subsequent leaf gas exchange measurements^63–65^.

### Leaf gas exchange measurements

Measurements of leaf gas exchange were made on the most recently fully expanded leaves during August to September 2015, between 9:00 and 12:00 h using the LI-6400 portable photosynthesis system (Li-Cor, Lincoln, Nebraska, USA). Maximum photosynthetic rate per unit area (*A*_max,a_) was determined for each species by measuring a light response curve (A-PPFD curves) at ambient 400 μmol mol^−1^ CO_2_, leaf temperature of 28–30 °C, and relative humidity of 40–60%, with the photosynthetic photon flux density (PPFD) order was 1500, 1200, 1000, 800, 500, 300, 200, 120, 100, 80, 50, 20,0 μmol m^−2^ s^−1^. For the photosynthesis measurement of masson pine needles, a bunch of needles were measured side by side, and then the average was calculated. Photosynthetic P-use efficiency (PPUE) was calculated as *A*_max_ divided by total P concentration (µmol CO_2_ mol P^−1^ s^−1^). *V*_cmax,a_ and *J*_max,a_ on an area basis were estimated from relationships between photosynthetic rate (*A*) and sub-stomatal CO_2_ mole fraction (*C*_*i*_)^28^ at fixed PPFD (1200 µmol photons m^−2^ s^−1^) following the CO_2_ order 400, 300, 200, 100, 50, 500, 700, 900, 1000, 1200, 1500 μmol mol^−1^.

### Leaf structure and nutrients

After photosynthetic measures, leaves with the same position as used for gas-exchange measurements were collected. Some of the leaves were immediately snap-frozen in liquid N and transferred on dry ice to laboratory. The samples were freeze-dried and stored at −80 °C until they were used for determination of foliar P fractions. The remaining leaves were oven-dried at 70 °C and then ground and homogenized for subsequent analyses. Total N concentration in dried leaves was determined using the Kjeldahl method. An additional sample of ten leaves was scanned to determine leaf area (LA) by LI-3000A portable system, dried at 70 °C for 72 h to a constant weight and measured for oven-dried mass (DM). Since the needles of masson pine trees don’t have flat leaf area, we record needle length and cross-section width and needle leaf area was estimated as: LA = π*L*D/2 (where LA is leaf area; L is needle length and D is needle width at the hale needle length). LMA, calculated as DM·LA^−1^ (g m^−2^), was used to calculate area-based nutrient concentrations (N_a_, P_a_; g m^−2^) from mass-based concentrations (N_m_, P_m_; mg g^−1^), and to shift area-based photosynthetic parameters (*A*_max,a_, *V*_cmax,a_, and *J*_max,a_; µmol CO_2_ m^−2^ s^−1^) to (*A*_max,m_, *V*_cmax,m_, and *J*_max,m_; µmol CO_2_ g^−1^ s^−1^). To compare LMA with mass-based leaf nutrient concentrations, we converted LMA into its reciprocal, leaf area per mass (SLA, cm^2^ g^−1^).

### Foliar P fractions

Foliar P fractions were extracted following methods outlined in Hidaka and Kitayama^37^. Each freeze-dried and ground sample (0.5 g DW) was homogenized and extracted twice with a total of 15 mL of 12:6:1 CMF (chloroform, methanol, and formic acid; v/v/v) and twice with a total of 19 mL of 1:2:0.8 CMW (chloroform, methanol, and water; v/v/v). Extracts were combined in a 50-mL centrifuge tube, and 9.5 mL of water was added to each tube. Thereafter, the extract was separated into an aqueous upper layer and a lipid-rich organic bottom layer. A subsample of the lipid layer was digested to give structural P. The residue was re-extracted for 1 h with 5 mL of 85% (w/v) methanol. The supernatant was added to the tube containing the aqueous layer. A 20 ml volume of the aqueous layer was added to another 50-ml graduated tube. The remaining aqueous layer was decanted into the tube containing the residue. The volume in the tube was increased to 20 ml with deionized water. After the preparation was cooled to 4 °C, 1 ml of cold 100% (w/v) trichloroacetic acid (TCA) was added to make a 5% (w/v) TCA solution. After extraction for 1 h, this cold TCA solution was subjected to a re-extraction with 10 mL of 5% (w/v) cold TCA 4 °C. A subsample of the supernatant was taken for the analysis of metabolic P. The residue was then re-extracted twice in a total of 35 mL of 2.5% (w⁄ v) TCA at 95 °C for 1 h. A subsample of the supernatant was taken for the analysis of nucleic acid P. The residue was digested to obtain residue P.

Each subsample was evaporated to dryness at 70 °C (organic solutions) or 100 °C (aqueous solutions) and digested for P determination as described above. The concentration of P in the digest was determined at 700 nm in a UV-Vis spectrophotometer (UV1800, Shimadzu, Japan) after a standard molybdate reaction. Total P concentration was the sum of foliar P fractions.

### Data analysis

To compare overall average leaf trait and foliar P fraction (concentration and percentage) values of the three successional groups using ANOVAs, and multiple comparisons were conducted using Duncan’s multiple range test. Bivariate regression was used to explore relationships between photosynthetic parameters and other leaf traits (N, P and LMA) among the three successional groups. Standardized major axis (SMA) regression was used to examine for variations in the slope and the y-axis intercept of bivariate leaf trait relationships, using SMATR version 2.0. software. When we tested the relationships among leaf traits, values were log_10_-transformed when it was necessary to normalize distributions. The significance of SMA regression was determined at the 0.05 level. We also used a linear mixed effects model combining fixed and random effects to account for variability in *V*_cmax_ and *J*_max_ on both area- and mass-bases. The model’s fixed effect included N, P and LMA (or SLA), and species as random effects. Model specification and validation were conducted in ‘R’ (version 3.4.0; R Development Core Team, 2011), using the nlme package. All of our data for the mixed-effect modelling analysis were log transformed. To select the best model, Akaike’s information criterion (AIC) was used. Statistical analysis was performed using SPSS 21.0 (IBM SPSS, USA), unless otherwise indicated.

## Electronic supplementary material

Supplementary Information

## References

[1] Elser JJ (2007). Global analysis of nitrogen and phosphorus limitation of primary producers in freshwater, marine and terrestrial ecosystems. Ecol. Lett..

[2] Vitousek PM, Porder S, Houlton BZ, Chadwick OA (2010). Terrestrial phosphorus limitation: mechanisms, implications, and nitrogen-phosphorus interactions. Ecol. Appl..

[3] Dalling J. W., Heineman K., Lopez, O. R., Joseph Wright, S. & Turner B. L. In *Tropical Tree Physiology*: adaptations and responses in a changing environment (eds Goldstein G. & Santiago L. S.) (Springer International Publishing, Switzerland 2016).

[4] Reich PB, Oleksyn J (2004). Global patterns of plant leaf N and P in relation to temperature and latitude. Proc. Natl. Acad. Sci. USA.

[5] Lambers H, Raven JA, Shaver GR, Smith SE (2008). Plant nutrient-acquisition strategies change with soil age. Trends Ecol. Evol..

[6] Domingues TF (2010). Co-limitation of photosynthetic capacity by nitrogen and phosphorus in West Africa woodlands. Plant Cell Environ..

[7] Koerselman W, Meuleman AFM (1996). The vegetation N:P ratio: A new tool to detect the nature of nutrient limitation. J. Appl. Ecol..

[8] Güsewell SN (2004). P ratios in terrestrial plants: variation and functional significance. New Phytol..

[9] Richardson SJ, Allen RB, Doherty JE (2008). Shifts in leaf N: P ratio during resorption reflect soil P in temperate rainforest. Funct. Ecol..

[10] Tessier JT, Raynal DJ (2003). Use of nitrogen to phosphorus ratios in plant tissue as an indicator of nutrient limitation and nitrogen saturation. J. Appl. Ecol..

[11] Dusenge ME (2015). Photosynthetic capacity of tropical montane tree species in relation to leaf nutrients, successional strategy and growth temperature. Oecologia.

[12] MacClendon JH (1962). The relationship between the thickness of deciduous leaves and their maximum photosynthetic rate. Am. J. Bot..

[13] Koike T (1988). Leaf structure and photosynthetic performance as related to the forest succession of deciduous broad-leaved trees. Plant Spec. Biol..

[14] Field, C. & Mooney, H. A. The photosynthesis-nitrogen relationship in wild plants. 25–55 (Cambridge University Press, 1986).

[15] Reich PB (1999). Generality of leaf trait relationships: a test across six biomes. Ecology.

[16] Poorter H, Niinemets U, Poorter L, Wright IJ, Villar R (2009). Causes and consequences of variation in leaf mass per area (LMA): a meta-analysis. New Phytol..

[17] Evans JR (1989). Photosynthesis and nitrogen relationship in leaves of C_3_ plants. Oecologia.

[18] Wright IJ (2004). The worldwide leaf economics spectrum. Nature.

[19] Denton MD, Veneklaas EJ, Freimoser FM, Lambers H (2007). Banksia species (Proteaceae) from severely phosphorus-impoverished soils exhibit extreme efficiency in the use and re-mobilization of phosphorus. Plant Cell Environ..

[20] Meir P, Levy PE, Grace J, Jarvis PG (2007). Photosynthetic parameters from two contrasting woody vegetation types in West Africa. Plant Ecol..

[21] Kattge J, Knorr W, Raddatz T, Wirth C (2009). Quantifying photosynthetic capacity and its relationship to leaf nitrogen content for global-scale terrestrial biosphere models. Glob. Change Biol..

[22] Domingues TF (2015). Biome-specific effects of nitrogen and phosphorus on the photosynthetic characteristics of trees at a forest-savanna boundary in Cameroon. Oecologia.

[23] Jacob J, Lawlor DW (1993). *In vivo* photosynthetic electron transport does not limit photosynthetic capacity in phosphate deficient sunflower and maize leaves. Plant Cell Environ..

[24] Campbell CD, Sage RF (2006). Interactions between the effects of atmospheric CO_2_ content and P nutrition on photosynthesis in white lupin (*Lupinus albus* L.). Plant Cell Environ..

[25] Hidaka A, Kitayama K (2009). Divergent patterns of photosynthetic phosphorus use efficiency versus nitrogen use efficiency of tree leaves along nutrient availability gradients. J. Ecol..

[26] Takashima T, Hikosaka K, Hirose T (2004). Photosynthesis or persistence: nitrogen allocation in leaves of evergreen and deciduous Quercus species. Plant Cell Environ..

[27] Reich PB, Oleksyn J, Wright IJ (2009). Leaf phosphorus influences the photosynthesis-nitrogen relation: a cross-biome analysis of 314 species. Oecologia.

[28] Farquhar GD, von Caemmerer S, Berry JA (1980). A biochemical model of photosynthetic CO_2_ assimilation in leaves of C_3_ species. Planta.

[29] Walker AP (2014). The relationship of leaf photosynthetic traits - *V*_cmax_ and *J*_max_ - to leaf nitrogen, leaf phosphorus, and specific leaf area: a meta-analysis and modeling study. Ecol. Evol..

[30] Raaimakers D, Boot RG, Dijkstra P, Pot S (1995). Photosynthetic rates in relation to leaf phosphorus content in pioneer versus climax tropical rainforest trees. Oecologia.

[31] Poorter L, Bongers F (2006). Leaf traits are good predictors of plant performance across 53 rain forest species. Ecology.

[32] Sobrado MA (2008). Leaf and photosynthetic characteristics of pioneer and forest species in tropical montane habitats. Photosynthetica.

[33] Valladares F, Niinemets U (2008). Shade tolerance, a key plant feature of complex nature and consequences. Annu. Rev. Ecol. Evol. S.

[34] Zhang Q (2015). Photosynthetic characteristics and light energy conversions under different light environments in five tree species occupying dominant status at different stages of subtropical forest succession. Funct. Plant Biol..

[35] Wardle DA, Walker LR, Bardgett RD (2004). Ecosystem properties and forest decline in contrasting long-term chronosequences. Science.

[36] Lambers H, Brundrett MC, Raven JA, Hopper SD (2010). Plant mineral nutrition in ancient landscapes: high plant species diversity on infertile soils is linked to functional diversity for nutritional strategies. Plant Soil..

[37] Kedrowski RA (1983). Extraction and analysis of nitrogen, phosphorus and carbon fractions in plant-material. J. Plant Nutr..

[38] Close DC, Beadle CL (2004). Total, and chemical fractions, of nitrogen and phosphorus in Eucalyptus seedling leaves: Effects of species, nursery fertiliser management and transplanting. Plant Soil..

[39] Hidaka A, Kitayama K (2011). Allocation of foliar phosphorus fractions and leaf traits of tropical tree species in response to decreased soil phosphorus availability on Mount Kinabalu, Borneo. J. Ecol..

[40] Hidaka A, Kitayama K (2013). Relationship between photosynthetic phosphorus-use efficiency and foliar phosphorus fractions in tropical tree species. Ecol. Evol..

[41] Han W, Fang J, Guo D, Zhang Y (2005). Leaf nitrogen and phosphorus stoichiometry across 753 terrestrial plant species in China. New Phytol..

[42] Liu X (2010). N and P stoichiometry of plant and soil in lower subtropical forest successional series in southern China. Chin. J. Plant Ecol..

[43] Hou E, Chen C, McGroddy ME, Wen D (2012). Nutrient limitation on ecosystem productivity and processes of mature and old-growth subtropical forests in China. PloS one.

[44] Huang WJ (2013). Increasing phosphorus limitation along three successional forests in southern China. Plant Soil..

[45] Rijkers T, Pons TL, Bongers F (2000). The effect of tree height and light availability on photosynthetic leaf traits of four neotropical species differing in shade tolerance. Func. Ecol..

[46] Craine JM, Morrow C, Stock WD (2008). Nutrient concentration ratios and co-limitation in South African grasslands. New Phytol..

[47] Norby RJ (2017). Informing models through empirical relationships between foliar phosphorus, nitrogen and photosynthesis across diverse woody species in tropical forests of Panama. New Phytol..

[48] Bloomfield KJ (2014). Contrasting photosynthetic characteristics of forest vs. savanna species (Far North Queensland, Australia). Biogeosciences.

[49] Sterner, R. W. & Elser, J. J. Ecological stoichiometry: the biology of elements from molecules to the biosphere (Princeton University Press, 2002).

[50] McGroddy ME, Daufresne T, Hedin LO (2004). Scaling of C:N:P stoichiometry in forests worldwide: implications of terrestrial redfield-type ratios. Ecology.

[51] van de Weg MJ, Meir P, Grace J, Atkin OK (2009). Altitudinal variation in leaf mass per unit area, leaf tissue density and foliar nitrogen and phosphorus content along an Amazon-Andes gradient in Peru. Plant Ecol. Divers..

[52] Whitehead D (2005). Photosynthesis and reflectance indices for rainforest species in ecosystems undergoing progression and retrogression along a soil fertility chronosequence in New Zealand. Oecologia.

[53] Terashima I, Miyazawa S, Hanba TY (2001). Why are sun leaves thicker than shade leaves? Consideration based on analyses of CO_2_ diffusion in the leaf. J. Plant Res..

[54] Niinemets Ü (2002). Stomatal conductance alone does not explain the decline in foliar photosynthetic rates with increasing tree age and size in *Picea abies* and *Pinus sylvestris*. Tree physiol..

[55] Wullschleger SD (1993). Biochemical limitations to carbon assimilation in C_3_ plants – a retrospective analysis of the *A/C*_*i*_ curves from 109 species. J. Exp. Bot..

[56] Medlyn BE (2002). Temperature response of parameters of a biochemically based model of photosynthesis. II. A review of experimental data. Plant Cell Environ..

[57] Bahar NH (2017). Leaf-level photosynthetic capacity in lowland Amazonian and high-elevation Andean tropical moist forests of Peru. New Phytol..

[58] Chapin FS, Kedrowski RA (1983). Seasonal changes in nitrogen and phosphorus fractions and autumn retranslocation in evergreen and deciduous taiga trees. Ecology.

[59] Villar R, Robleto JR, De Jong Y, Poorter H (2006). Differences in construction costs and chemical composition between deciduous and evergreen woody species are small as compared to differences among families. Plant Cell Environ..

[60] Chapin FS, Shaver GR, Kedrowski RA (1986). Environmental controls over carbon, nitrogen and phosphorus fractions in *Eriophorum Vaginatum* in Alaskan Tussock Tundra. J. Ecol..

[61] Zhang Q. M. Dataset of China Ecosystem Research Network: Dinghushan Forest Ecosystem Research Station (1998–2008). (China Agriculture Press, Beijing 2011).

[62] Zhu SD, Song JJ, Li RH, Ye Q (2013). Plant hydraulics and photosynthesis of 34 woody species from different successional stages of subtropical forests. Plant Cell Environ..

[63] Ellsworth DS, Reich PB (1993). Canopy structure and vertical patterns of photosynthesis and related leaf traits in a deciduous forest. Oecologia.

[64] Kolb TE, Stone JE (2000). Differences in leaf gas exchange and water relations among species and tree sizes in an Arizona pine-oak forest. Tree physiol..

[65] Wyka TP (2012). Responses of leaf structure and photosynthetic properties to intra-canopy light gradients: a common garden test with four broadleaf deciduous angiosperm and seven evergreen conifer tree species. Oecologia.

