# Supplementary material for: Photosynthesis of subtropical forest species from different successional status in relation to foliar nutrients and phosphorus fractions
Source: Sci Rep. 2018 Jul 11;8:10455. doi: 10.1038/s41598-018-28800-4 (PMC6041293; doi:10.1038/s41598-018-28800-4)
Supplement: Supplementary file 1 — Supplementary Information [file 41598_2018_28800_MOESM1_ESM.docx]

**Photosynthesis of subtropical forest species from different successional status in relation to foliar nutrients and phosphorus fractions**

Guihua Zhang^1,2,^^+^, Lingling Zhang^1,3, +^ & Dazhi Wen^1,3,*^

^1^Key Laboratory of Vegetation Restoration and Management of Degraded Ecosystems, South China Botanical Garden, Chinese Academy of Sciences, Guangzhou 510650, China; ^2^ College of Resources and Environment, University of Chinese Academy of Sciences, Beijing 100049, China; ^3^Guangdong Provincial Key Laboratory of Applied Botany, South China Botanical Garden, Chinese Academy of Sciences, Guangzhou 510650, China.

**Supplementary Information**

Supplementary Figures S1

Supplementary Tables S1–S6

**
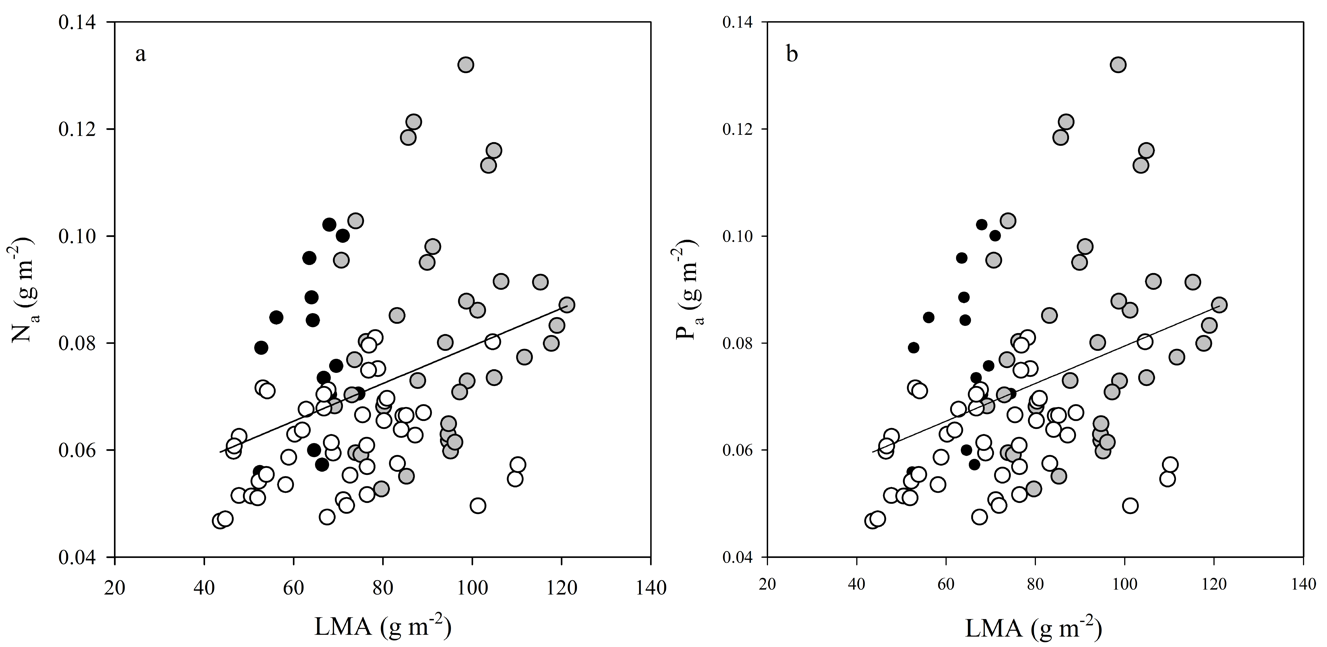
**

**Supplementary Figure S1**. The relationships between log-log area-based leaf nitrogen concentration (N_a_), leaf phosphorus concentration (P_a_), and leaf mass per area (LMA). Data points represent individual leaf values (19, 37, and 47 individuals from pioneer, generalist, and climax species, respectively). Standardized major axis (SMA) regressions are given in Supplementary Table S2. Symbols: pioneer species (solid); generalist species (grey); climax species (open).

**Supplementary Table S1**. Mass-based leaf traits for the subtropical forest species in three successional groups

| Group | N_m_  (mg g^-1^) | P_m_  (mg g^-1^) | N:P ratio | SLA  (cm^2^ g^-1^) | *A*_max,m_  (µmol g^-1^ s^-1^) | *V*_cmax,m_  (µmol g^-1^ s^-1^) | *J*_max,m_  (µmol g^-1^ s^-1^) | *J*_max,m_/*V*_cmax,m_ |
| --- | --- | --- | --- | --- | --- | --- | --- | --- |
| Pioneer | 21.56 ± 1.74 b | 1.18 ± 0.05 b | 18.28 ± 1.40 a | 133.59 ± 9.77 b | 0.17 ± 0.02b | 0.88 ± 0.07 b | 1.11 ± 0.08 b | 1.29 ± 0.05 a |
| Generalist | 17.38 ± 0.52 a | 0.90 ± 0.04 a | 19.99 ± 0.69 a | 110.67 ± 2.87 a | 0.11 ± 0.01a | 0.63 ± 0.04 a | 0.82 ± 0.05 a | 1.31 ± 0.04 a |
| Climax | 21.59 ± 0.97 b | 0.91 ± 0.03 a | 23.92 ± 0.88 b | 150.27 ± 5.39 b | 0.10 ± 0.01a | 0.75 ± 0.04 ab | 0.91 ± 0.04 a | 1.25 ± 0.03 a |

Values shown are group averages (± SE). Means in a column followed by different letters are significantly different (*P* < 0.05). N_m_, leaf nitrogen concentration; P_m_, leaf phosphorus concentration; leaf N: P ratio, leaf nitrogen to phosphorus ratio; SLA, leaf area per mass; *A*_max,m_, maximum photosynthesis assimilate rate; *V*_cmax,m_, maximum carboxylation velocity; *J*_max,m_, maximum electron transport rate.

**Supplementary Table S2**. Standardized major axis regression slopes and their confidence intervals for log–log transformed relationships comparing leaf traits of pioneer species (5 species, 19 individuals), generalist species (8 species, 37 individuals), and climax species (11 species, 47 individuals). Analysis undertaken using individual replicates of area-based. Coefficients of determination (r^2^) and significance values (*P*) of each bivariate relationship are also shown. 95% confidence intervals (CI) of SMA slopes and y-axis intercepts are shown in parentheses. Where SMA tests for common slopes revealed no significant differences between the three successional strategy groups (i.e., *P* > 0.05), common slopes were used (with CI of the common slopes provided). If there was a significant difference in the elevation (i.e., the y-axis intercept) of the common-slope SMA regressions, values for the y-axis intercept are provided. Where appropriate, significant shifts along a common slope are indicated. Na, leaf nitrogen concentration; Pa, leaf phosphorus concentration; LMA, leaf mass per area

| Bivariate relationship  (y-vs-x axis) | Group | r^2^ | *p* | Slope | Slope Cl | Intercept | *p* | Common slope | Common slope CI | *p* | common slope *y*-axis intercept | Shift along a  common slope? |
| --- | --- | --- | --- | --- | --- | --- | --- | --- | --- | --- | --- | --- |
| N_a_ vs LMA | Pioneer | 0.142 | 0.112 | 0.616 | (0.390, 0.974) | -0.964 | 0.080 | 0.983 | (0.813, 1.203) | 0.004 | -1.662 | Yes, *p* < 0.001 |
|  | Generalist | 0.119 | 0.037 | 1.107 | (0.807, 1.520) | -1.978 |  |  |  |  | -1.734 |  |
|  | Climax | 0.091 | 0.039 | 1.071 | (0.807, 1.420) | -1.815 |  |  |  |  | -1.654 |  |
| P_a_ vs LMA | Pioneer | 0.778 | <0.001 | 0.935 | (0.736, 1.187) | -2.811 | 0.002 | 0.914 |  |  |  |  |
|  | Generalist | 0.054 | 0.167 | 1.498 | (1.079, 2.079) | -4.035 |  |  |  |  |  |  |
|  | Climax | 0.101 | 0.029 | 0.613 | (0.463, 0.812 | -2.34 |  |  |  |  |  |  |

**Supplementary** **Table S3**. Standardized major axis regression slopes and their confidence intervals for log–log transformed relationships comparing leaf traits of pioneer species (5 species, 19 individuals), generalist species (8 species, 37 individuals), and climax species (11 species, 47 individuals). Analysis undertaken using individual replicates of area- and mass-based. Coefficients of determination (*r*2) and significance values (*P*) of each bivariate relationship are shown. 95% confidence intervals (CI) of SMA slopes and *y-*axis intercepts are shown in parentheses. If SMA tests for common slopes revealed no significant differences (i.e., *P* > 0.05) among the three successional groups, common slopes were used (with CI of the common slopes provided). If there was a significant difference in the elevation (i.e., *y*-axis intercept) of the common-slope SMA regressions, values for the *y*-axis intercept are provided. Where appropriate, significant shifts along a common slope are indicated. N, leaf nitrogen concentration; P, leaf phosphorus concentration; LMA, leaf mass per area; *A*_max_, maximum photosynthesis assimilate rate

| Bivariate relationship  (*y* vs. *x* axis) | Group | r^2^ | *p* | Slope | Slope Cl | Intercept | *p* | Common slope | Common slope Cl | *p* | Common slope *y*-axis intercept | Shift along a common slope? |
| --- | --- | --- | --- | --- | --- | --- | --- | --- | --- | --- | --- | --- |
| *A*_max,m_ vs N_m_ | Pioneer | 0.647 | <0.001 | 1.391 | (1.031, 1.876) | -2.649 | 0.052 | 1.533 | (1.295, 1.826) | <0.001 | -2.835 | Yes, *p* = 0.048 |
|  | Generalist | 0.139 | 0.023 | 2.165 | (1.583, 2.961) | -3.659 |  |  |  |  | -2.880 |  |
|  | Climax | 0.218 | 0.001 | 1.319 | (1.015, 1.716) | -2.775 |  |  |  |  | -3.056 |  |
| *A*_max,m_ vs P_m_ | Pioneer | 0.012 | 0.650 | 2.804 | (1.720, 4.571) | -1.016 | 0.127 | 1.728 | (1.468, 2.203) | 0.301 | -0.946 | Yes, *p* < 0.001 |
|  | Generalist | 0.055 | 0.163 | 1.640 | (1.182, 2.277) | -0.893 |  |  |  |  | -0.888 |  |
|  | Climax | 0.571 | <0.001 | 1.627 | (1.338, 1.978) | -0.958 |  |  |  |  | -0.953 |  |
| *A*_max,m_ vs LMA | Pioneer | 0.480 | 0.001 | -1.331 | (-1.909, -0.928) | 1.698 | 0.010 | -1.798 |  |  |  |  |
|  | Generalist | 0.282 | 0.001 | -2.622 | (-3.493, -1.969) | 4.152 |  |  |  |  |  |  |
|  | Climax | 0.357 | <0.001 | -1.615 | (-2.050, -1.272) | 1.924 |  |  |  |  |  |  |
| *A*_max,a_ vs N_a_ | Pioneer | 0.317 | 0.012 | 1.562 | (1.036, 2.357) | 0.745 | 0.09 | 1.525 | (1.249, 1.853) | <0.001 | 0.752 | Yes, *p* < 0.001 |
|  | Generalist | 0.005 | 0.683 | 2.035 | (1.454, 2.848) | 0.576 |  |  |  |  | 0.675 |  |
|  | Climax | 0.041 | 0.172 | 1.21 | (0.905, 1.616) | 0.613 |  |  |  |  | 0.565 |  |
| *A*_max,a_ vs P_a_ | Pioneer | 0.001 | 0.902 | -1.029 | (-1.682, -0.629) | 0.006 | 0.014 | 1.714 |  |  |  |  |
|  | Generalist | <0.001 | 0.912 | -1.504 | (-2.106, -1.074) | -0.681 |  |  |  |  |  |  |
|  | Climax | 0.308 | 0.001 | 2.114 | (1.651, 2.706) | 3.363 |  |  |  |  |  |  |
| *A*_max,a_ vs LMA | Pioneer | 0.006 | 0.743 | 0.962 | (0.590, 1.571) | -0.761 | 0.007 | -1.498 |  |  |  |  |
|  | Generalist | 0.030 | 0.302 | -2.256 | (-3.144, -1.619) | 5.397 |  |  |  |  |  |  |
|  | Climax | 0.001 | 0.858 | 1.295 | (0.963, 1.741) | -1.581 |  |  |  |  |  |  |

**Supplementary Table S4**. Standardized major axis regression slopes and their confidence intervals for log–log transformed when necessary relationships comparing leaf traits of pioneer species (5 species, 19 individuals), generalist species (8 species, 37 individuals), and climax species (11 species, 47 individuals). Analysis undertaken using individual replicates of area-based. Coefficients of determination (*r*2) and significance values (*P*) of each bivariate relationship are also shown. 95% confidence intervals (CI) of SMA slopes and *y-*axis intercepts are shown in parentheses. Where SMA tests for common slopes revealed no significant differences between the three successional strategy groups (i.e., *P* > 0.05), common slopes were used (with CI of the common slopes provided). If there was a significant difference in the elevation (i.e., the *y*- axis intercept) of the common-slope SMA regressions, values for the *y*-axis intercept are provided. Where appropriate, significant shifts along a common slope are indicated. N_a_, leaf nitrogen concentration; P_a_, leaf phosphorus concentration; leaf N: P, leaf nitrogen to phosphorus ratio; LMA, leaf mass per area; *V*_cmax,a_, maximum carboxylation velocity; *J*_max,a_, maximum electron transport rate

| Bivariate relationship  (y-vs-x axis) | Group | r^2^ | *p* | Slope | Slope Cl | Intercept | *p* | Common slope | Common slope CI | *p* | common slope *y*-axis intercept | Shift along a  common slope? |
| --- | --- | --- | --- | --- | --- | --- | --- | --- | --- | --- | --- | --- |
| *J*_max,a_ vs *V*_cmax,a_  not log-transformed | Pioneer | 0.697 | <0.001 | 1.504 | (1.139, 1.985) | -15.150 | 0.372 | 1.272 | (1.133, 1.421) | 0.481 | 0.860 | Yes, *p* < 0.001 |
|  | Generalist | 0.729 | <0.001 | 1.256 | (1.052, 1.501) | 1.821 |  |  |  |  | 0.946 |  |
|  | Climax | 0.672 | <0.001 | 1.201 | (1.012, 1.425) | 1.370 |  |  |  |  | -2.133 |  |
| *V*_cmax,a_ vs LMA | Pioneer | 0.274 | 0.021 | 0.810 | (0.530, 1.236) | 0.275 | 0.003 | 1.338 |  |  |  |  |
|  | Generalist | 0.000 | 0.964 | 2.052 | (1.466, 2.874) | -2.291 |  |  |  |  |  |  |
|  | Climax | 0.010 | 0.503 | -1.238 | (-1.662, -0.922) | 3.946 |  |  |  |  |  |  |
| *V*_cmax,a_ vs N_a_ | Pioneer | 0.043 | 0.396 | 1.314 | (0.812, 2.128) | 1.542 | 0.071 | 1.427 | (1.174, 1.730) | 0.404 | 1.519 | Yes, *p* = 0.001 |
|  | Generalist | 0.239 | 0.002 | 1.854 | (1.380, 2.490) | 1.375 |  |  |  |  | 1.457 |  |
|  | Climax | 0.083 | 0.050 | 1.156 | (0.871, 1.536) | 1.499 |  |  |  |  | 1.458 |  |
| *V*_cmax,a_ vs P_a_ | Pioneer | 0.155 | 0.095 | 0.866 | (0.550, 1.365) | 2.710 | 0.004 | 1.502 |  |  |  |  |
|  | Generalist | 0.075 | 0.100 | 1.371 | (0.991, 1.896) | 3.329 |  |  |  |  |  |  |
|  | Climax | 0.064 | 0.085 | 2.021 | (1.517, 2.691) | 4.128 |  |  |  |  |  |  |
| *V*_cmax,a_ vs leaf N:P | Pioneer | 0.086 | 0.222 | -0.992 | (-1.589, -0.619) | 3.045 | 0.220 | 1.296 | (1.591, 1.063) | <0.001 | 0.207 | No, *p* = 0.737 |
|  | Generalist | 0.010 | 0.550 | 1.624 | (1.162, 2.270) | -0.364 |  |  |  |  | 0.059 |  |
|  | Climax | 0.023 | 0.312 | 1.221 | (0.911, 1.635) | 0.007 |  |  |  |  | -0.097 |  |
| *J*_max,a_ vs LMA | Pioneer | 0.256 | 0.027 | 1.067 | (0.695, 1.637) | -0.120 | 0.017 | 1.371 |  |  |  |  |
|  | Generalist | 0.003 | 0.751 | -2.025 | (-2.645, -1.358) | 5.809 |  |  |  |  |  |  |
|  | Climax | 0.000 | 0.948 | 1.164 | (0.866, 1.565) | -0.375 |  |  |  |  |  |  |
| *J*_max,a_ vs N_a_ | Pioneer | 0.064 | 0.296 | 1.732 | (1.075, 2.790) | 1.549 | 0.033 | 1.442 |  |  |  |  |
|  | Generalist | 0.273 | 0.001 | 1.829 | (1.370, 2.441) | 1.483 |  |  |  |  |  |  |
|  | Climax | 0.169 | 0.004 | 1.087 | (0.830, 1.425) | 1.598 |  |  |  |  |  |  |
| *J*_max,a_ vs P_a_ | Pioneer | 0.194 | 0.059 | 1.141 | (0.732, 1.780) | 3.088 | 0.103 | 1.506 | (1.247, 1.842) | 0.015 | 3.465 | Yes, *p* < 0.001 |
|  | Generalist | 0.170 | 0.011 | 1.352 | (0.994, 1.840) | 3.323 |  |  |  |  | 3.492 |  |
|  | Climax | 0.032 | 0.232 | 1.900 | (1.420, 2.543) | 4.071 |  |  |  |  | 3.592 |  |
| *J*_max,a_ vs leaf N:P | Pioneer | 0.098 | 0.191 | -1.307 | (-2.088, -0.818) | 3.530 | 0.331 | 1.315 | (1.600, 1.080) | <0.001 | 0.277 | No, *p* = 0.886 |
|  | Generalist | 0.001 | 0.851 | -1.602 | (-2.243, -1.144) | 3.908 |  |  |  |  | 0.138 |  |
|  | Climax | 0.106 | 0.025 | 1.148 | (0.867, 1.519) | 0.195 |  |  |  |  | -0.034 |  |

**Supplementary Table S5**. Stepwise selection process for the fixed component of linear mixed effect models: with *V*_cmax_ and *J*_max_ as the response variables. The best predictive models, underlined, were presented in Table 2. For the best models, explanatory variables are: leaf nitrogen (N) and phosphorus (P) concentrations; leaf area per mass (SLA) and leaf mass per area (LMA). Species was used as a random component of the model. Models variants were all run using the Maximum Likelihood method; the model's random component was identical in all variants. Test parameters and statistics are DF (degrees of freedom), AIC (Akaike Information Criterion), and logLik (maximum Likelihood).

|  | *V*_cmax_ | DF | AIC | logLik |  | *J*_max_ | DF | AIC | logLik |
| --- | --- | --- | --- | --- | --- | --- | --- | --- | --- |
| (a) Mass-based model | |  |  |  |  |  |  |  |  |
| All | -0.71+0.5*Pm +0.21*Nm*SLA | 5 | -127.15 | 68.57 |  | -0.56+0.48*Pm + 0.19*Nm*SLA | 5 | -145.19 | 77.59 |
| Pioneer | -1.36+0.61*SLA | 4 | -19.88 | 13.94 |  | -0.45+0.17*N_m_*SLA | 4 | -20.12 | 14.06 |
| Generalist | -1.44+0.48N_m_ * SLA | 4 | -43.02 | 25.52 |  | -1.42+0.52*N_m_*SLA | 4 | -50.61 | 29.31 |
| Climax | -0.66+0.63*P_m_ +0.19*N_m_*SLA | 5 | -59.13 | 34.56 |  | -0.89+0.29*N_m_*SLA | 4 | -71.26 | 39.63 |
| (b) Area-based model | |  |  |  |  |  |  |  |  |
| All | 2.22+0.33*N_a_ +0.26* P_a_*LMA | 5 | -128.98 | 69.49 |  | 1.72+2.67*N_a_ +1.90* N_a_*P_a_ | 5 | -149.91 | 79.95 |
| Pioneer | 1.08+0.39*LMA | 4 | -16.11 | 13.94 |  | 1.00+0.49*LMA | 4 | -19.67 | 13.83 |
| Generalist | 2.34+0.28*P_a_ * LMA | 4 | -39.92 | 23.96 |  | 3.08-0.74*LMA+0.55*N_a_*LMA | 5 | -48.61 | 29.3 |
| Climax | 1.66+6.18*N_a_ - 3.26*N_a_*LMA | 5 | -60.67 | 35.34 |  | 1.70+0.44*N_a_ | 4 | -72.68 | 40.34 |

**Supplementary Table S6**. Characteristics of the 24 forest species investigated in this study

| Species name | Family name | Successional group | No. individuals per species |
| --- | --- | --- | --- |
| *Pinus massoniana* | Pinaceae | Pioneer | 5 |
| *Evodia lepta* | Rutaceae | Pioneer | 4 |
| *Mallotus paniculatus* | Euphorbiaceae | Pioneer | 4 |
| *Melastoma candidum* | Melastomataceae | Pioneer | 3 |
| *Pterospermum heterophyllum* | Sterculiaceae | Pioneer | 3 |
| *Aporosa chinensis* | Euphorbiaceae | Generalist | 4 |
| *Castanopsis chinensis* | Fagaceae | Generalist | 5 |
| *Castanopsis fissa* | Fagaceae | Generalist | 5 |
| *Cryptocarya chinensis* | Lauraceae | Generalist | 5 |
| *Machilus chinensis* | Lauraceae | Generalist | 5 |
| *Psychotria rubra* | Rubiaceae | Generalist | 3 |
| *Schefflera octophylla* | Araliaceae | Generalist | 5 |
| *Schima superba* | Theaceae | Generalist | 5 |
| *Acmena acuminatissima* | Myrtaceae | Climax | 5 |
| *Aporusa yunnanensis* | Euphorbiaceae | Climax | 5 |
| *Aidia canthioides* | Rutaceae | Climax | 5 |
| *Engelhardtia roxburghiana* | Juglandaceae | Climax | 4 |
| *Gironniera subaequalis* | Ulmaceae | Climax | 5 |
| *Memecylon ligustrifolium* | Melastomataceae | Climax | 3 |
| *Microdesmis caseariifolia* | Pandaceae | Climax | 3 |
| *Pygeum topengii* | Rosaceae | Climax | 5 |
| *Sterculia lanceolata* | Sterculiaceae | Climax | 5 |
| *Syzygium rehderianum* | Myrtaceae | Climax | 4 |
| *Xanthophyllum hainanense* | Polygalaceae | Climax | 3 |
